# Magnetoresistance in the Spin-Orbit Kondo State of Elemental Bismuth

**DOI:** 10.1038/srep13772

**Published:** 2015-09-11

**Authors:** Luis Craco, Stefano Leoni

**Affiliations:** 1 Instituto de F sica, Universidade Federal de Mato Grosso, 78060-900, Cuiabá, MT, Brazil; 2 School of Chemistry, Cardiff University, Cardiff, CF10 3AT, UK

## Abstract

Materials with strong spin-orbit coupling, which competes with other particle-particle interactions and external perturbations, offer a promising route to explore novel phases of quantum matter. Using LDA + DMFT we reveal the complex interplay between local, multi-orbital Coulomb and spin-orbit interaction in elemental bismuth. Our theory quantifies the role played by collective dynamical fluctuations in the spin-orbit Kondo state. The correlated electronic structure we derive is promising in the sense that it leads to results that might explain why moderate magnetic fields can generate Dirac valleys and directional-selective magnetoresistance responses within spin-orbit Kondo metals.

Elemental bismuth, a material with a strong spin-orbit (SO) interaction, plays a key role in solid-state physics^1^ and in the history of metals^2^,^3^,^4^. During the last two decades, it has been investigated with respect to both its fundamental properties^5^,^6^,^7^,^8^,^9^,^10^ and its possible applications^11^,^12^. Its one-electron band structure displays an indirect negative band gap between the valence band maximum and the conduction band minima^13^. Because of the small valence and conduction band overlap bismuth is semimetal with a reduced number of charge carriers at the Fermi energy, *E*_*F*_. Beyond the inherent importance of exploring complex phases of quantum matter^5^,^6^, semimetal Bi is a material of great interest for technological applications involving use in spintronic devices^14^ and in magnetic field sensing^15^. Of particular interest for technological applications is the magnetoresistance (MR) effect^16^ which persists up to room temperature (*T*)^11^.

During the 20th century, transport properties of bismuth were extensively studied^1^,^4^. In the early 1930s, it was discovered that the resistivity of bismuth crystals increases by several orders of magnitude in the presence of external magnetic fields^2^,^3^. This remarkable phenomenon gives rise to large MR effects^17^. In fact, *P*-band semimetals like bulk bismuth and graphite undergo a metal-insulator-like transition upon exposure to modest magnetic fields^18^. The MR phenomena is believed to originate from an extremely small Fermi surface, very low carrier concentrations, long carrier mean-free paths and small effective fermion (electron/hole) masses. Resistivity studies also suggest that the Fermi liquid (FL) *T*^2^ dependence works quite well down to 4 K^19^. However, a detailed low-*T* study showed significant deviation from canonical FL systems, where the *T*^2^ behaviour changes smoothly into a *T*^5^-like form at very low temperatures^20^. Microscopic quantum theories involving the coupling between electrons and phonons^21^ or electron and holes (with different masses)^22^ have been proposed to explain this exotic low-*T* behavior. Complex low-energy physics in bismuth is also unveiled by optical measurements, showing large changes in plasma frequency and anomalous midinfrared features^23^,^24^. When considered altogether, these data are compelling evidence of unconventional electronic degrees of freedom in bismuth. Despite many years of research effort, it is of prime importance to revisit its bulk electronic structure and transport properties in view of understanding the intrinsic nature of competing quantum fluctuations in this system. What is the source of the electronic excitations in this rhombohedral one-band semimetal? Why is resistivity drastically enhanced by the application of moderate magnetic fields?

Deviations from FL-like behaviour^6^,^11^,^18^,^20^ and Hall coefficient measurements suggest that electronic correlations in bismuth may require serious consideration as this element may host an exotic quantum liquid^5^. In semimetals, where charge carriers interact via Coulomb interactions^25^ a treatment of dynamical correlations is required. Accordingly, self-consistent dynamical mean-field theory (DMFT) calculations in combination with local-density approximation (LDA) were performed. LDA + DMFT has proven to be the state-of-the-art method of choice for correlated materials^26^. In Bi-chalcogenide *p*-band topological insulators, we have recently shown that Kondo- and Mott-like features are well accounted for by the diagrammatic multi-orbital (MO) iterated-perturbation-theory^27^,^28^, which requires incorporation of sizeable MO Hubbard and SO interactions. As in the case of graphite^29^, here we demonstrate that a MO Kondo scenario also emerges for elemental bismuth, owing to their common layered character. If bismuth is considered a moderately correlated semimetal (with *U*_*eff*_  ≡ *U*/*W*_*LDA*_ ≈ 1, *U* and *W*_*LDA*_ are, respectively, the on-site Coulomb repulsion and the LDA band width) good semiquantitative accord with experimental data can be obtained. MO-Hubbard plus SO interaction qualitatively account for dynamical signatures of bulk correlation effects in photoemission (PES)^6^, inverse-PES^30^ and resistivity data^6^,^11^,^18^, revealing their intrinsic dynamical quantum nature.

The possibility of correlated electron physics in purely *p*^31^,^32^,^33^,^34^,^35^ or *s*^36^ band systems is very intriguing, since the naive expectation dictates that the itinerance (kinetic energy of *p*, *s*-carriers) is appreciable compared to the electron-electron interactions, as distinct from *d*-band systems, where the *d* electrons reside in much narrower bands (hence the effective *U*/*W*_*LDA*_ is sizable). However, we recall here that in canonical *s* metals like elemental lithium evidences for correlated electron physics have been reported by Stutz *et al.*^37^. Interestingly, a possible explanation for the breakdown of the FL picture observed in high-resolution Compton scattering experiments of bulk Li^37^ were ascribed to the possible existence of significant resonating-valence-bond (RVB) pairing correlation effects in the ground state of Li nanoclusters^38^. An additional interesting case of fundamental importance is given by Li impurities embedded aluminium, showing significantly deviations from a conventional FL picture due to interplay between alloy disorder and electronic correlations in the ground state of Al_97_Li_3_^39^. Thus, understanding the role of electron-electron interactions in materials with active *p* or *s* bands is undoubtedly an issue of great contemporary interest. In light of the discussion above, we study how an orbital-selective interplay between appreciable *p*-band itinerance and on-site Coulomb repulsion, *U*, plays a central role in this unique spin-orbit Kondo state of elemental bismuth.

## Results

**Figure 1:**
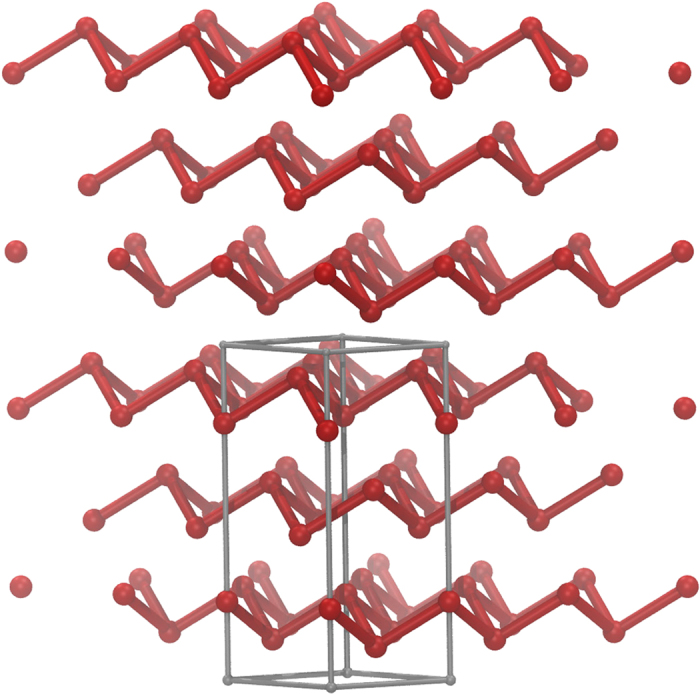
Crystal structure of Bismuth of A7, *α*-arsenic type structure, in the hexagonal setting. The unit cell is represented as a grey box, with the layer stacking developing along [001].

**Figure 2:**
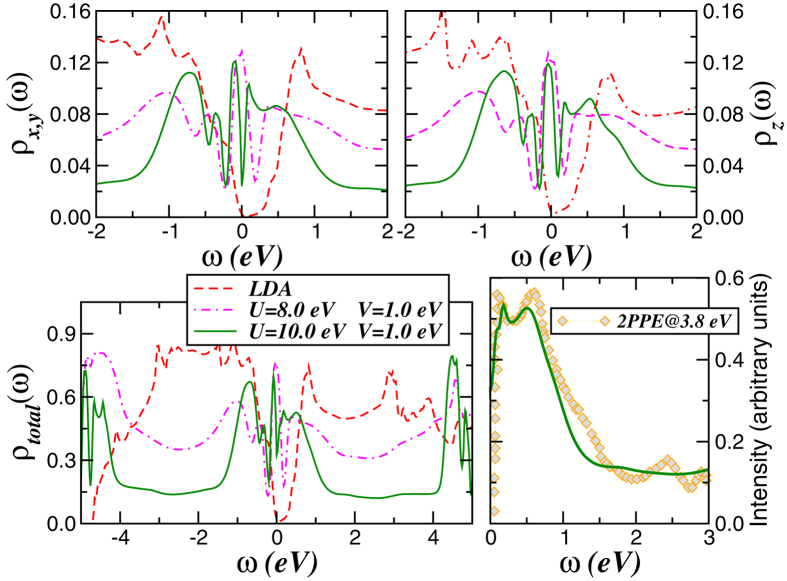
Spectral functions and two-photon photoemission. LDA (dashed) and LDA + DMFT [for *U* = 8.0 eV (dot-dashed), *U* = 10.0 eV (solid) and fixed *J*_*H*_ = 0.5 eV] orbital-resolved and total density-of-states (DOS) of bismuth. Notice the band gap near the Fermi energy (*E*_*F*_ = *ω* = 0) in LDA and bulk metallicity in the LDA + DMFT results. Right-lower panel: Comparison between the LDA + DMFT total DOS and two-photon photoemission (2PPE) data taken at *T* = 110 K for Bi(111)^30^. The 2PPE curve is shifted downward to coincide with theory at low energies. As seen, the low-energy peaks are accurately resolved within LDA + DMFT.

Bismuth crystallises in the *A*7, *α*-arsenic type structure (Fig. 1). In the rhombohedral setting, the unit cell contains two atoms. As homologue P and As, A7 Bi is characterised by extended puckered layers of three-connected Bi atoms, with shorter distances within each layer than between (111) layers. In Bi however, the difference between the two sets of distances is reduced, such that layered A7 Bi is better viewed as a distorted primitive simple cubic (PSC) structure^40^. Simple cubic bismuth would have a partially filled valence band, and would accordingly be metallic^1^,^40^. Upon rhombohedral distortion, A7 Bi becomes a semimetal with a small number of valence band states at the Fermi energy (*E*_*F*_), see Fig. 2. Here, LDA calculations for the real crystal structure at ambient conditions were performed using the linear muffin-tin orbitals (LMTO)^41^,^42^ scheme in the atomic sphere approximation. The corresponding orbital resolved and total LDA density-of-states (DOS) are shown in Fig. 2. In good agreement with previous studies^43^, the total DOS shows a tiny one-electron band gap centred at 0.035 eV above *E*_*F*_. Elemental bismuth has a formal +3 oxidation state, which implies an equal number of electrons and holes in the *p* sector. As pointed out, all electronic 6*p* states acquire some itinerance, providing valence and conduction band states in all active 6*p*-(*x*, *y*, *z*) orbitals. The situation encountered here with three half-filled bands provides the underlying microscopic one-band seeds of a quantum correlated scenario in elemental bismuth.

Within LDA, the one-electron part of the many-body Hamiltonian for bismuth is 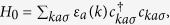 where *a* = *x*, *y*, *z* labels the diagonalised 6*p*-bands. In light of the sizable correlation effects within the Bi-channel of bulk Bi_2_Se_3_ and Bi_2_Te_2_Se topological insulators^27^,^28^, local MO Hubbard plus SO interactions must be included to describe the onset of correlated spectral functions. These constitute the interaction term 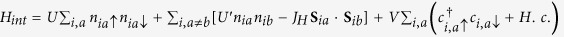. Here, *U*′ ≡ *U* − 2*J*_*H*_ and *U*(*U*′) is the intra- (inter-) orbital Coulomb repulsion. *J*_*H*_ and *V* are, respectively, the Hund’s rule and the local SO coupling. Within our formulation, the SO interaction acts as a transverse magnetic-field^44^ and locally mixes the *p*_*a*_ spin states of bismuth. The DMFT self-energy, Σ_*a*_(*ω*), requires a solution of the MO quantum impurity problem self-consistently embedded in an effective medium^26^. We use the multi-orbital iterated-perturbation-theory (MO-IPT) as an impurity solver for DMFT^45^: This perturbative, many-particle scheme has a proven record of successes in describing finite temperature effects^46^ and unconventional electronic transitions induced by local quantum fluctuations in correlated electron systems^47^.

Electron-electron interactions may induce topologically nontrivial electronic phases that can change the nature of single-particle excitation of topological insulators^48^,^49^. We have recently shown that the on-site Coulomb repulsion *U* promotes a gradual reduction of the bulk band gap size and coherent Kondo clouds are created near *E*_*F*_ in Bi-chalcogenide topological insulators^27^,^28^. Additionally, by further increasing *U* the energy gap is suppressed by dynamic transfer of spectral weight characteristic of correlated electron systems^50^,^51^ and renormalised quasiparticles are formed at low energies in Bi_2_*Y*_2_*X* (*Y* = (Se, Te), *X* = Se) topological materials. In Fig. 2 we display our LDA + DMFT results for fixed *J*_*H*_ = 0.5 eV^27^,^28^ and total band filling *n*_*t*_ = 3.0, showing that a quantum correlated scenario^5^ is applicable to bismuth. Several interesting features compared to LDA are manifested in Fig. 2: A combined effect of local, SO and electron-electron interactions arising from *U*, *U*′ and *J*_*H*_ leads to spectral weight redistribution over large energy scales and the formation of an electronic structure similar to topological Kondo systems^27^,^28^, with concomitant appearance of quasiparticle resonances at low-energies. The most salient features to be seen in Fig. 2 are the changes in the electronic states at low energies, where the emergent *spin-orbit Kondo* state is characterised by the presence of sharp in-gap states near the Fermi energy, *E*_*F*_(= *ω* = 0). As a first testing ground to our proposal, in Fig. 2 we compare our LDA + DMFT results to a recent experiment probing the unoccupied electronic states of bismuth. Very good semiquantitative agreement with two-photon photoemission (2PPE) data on Bi(111)^30^ is visible. Particularly, the low-energy peaks at energies below 0.6 eV as well as the spectral lineshape of the conduction band states up to 3 eV are accurately described by LDA + DMFT. The good agreement in a rather wide energy region is the stringent signature of spectral weight transfer (SWT) of correlated electrons.

**Figure 3:**
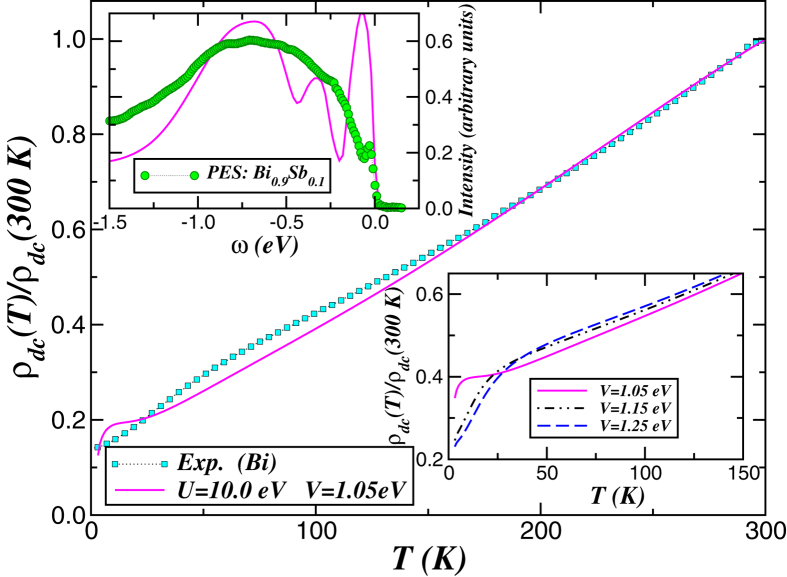
dc resistivity and photoemission. Main panel: Theory-experiment comparison of dc resistivity [*ρ*_*dc*_(*T*)] of bismuth, showing good semiquantitative agreement with experiment^6^ above 30 K. Notice the quasilinear temperature dependence of transport data which is quantitatively reproduced by LDA + DMFT. (Noteworthy, the theory curve was rescaled by an overall constant to coincide with experiment at 300 K.) Right-lower panel: Temperature dependence of dc resistivities as function of *V*: Interesting are the changes in slope of *ρ*_*dc*_(*T*) below 33 K with increasing *V* in good semiquantitative agreement with experiments^11^,^18^. Left-upper panel: Comparison between the LDA + DMFT total DOS and photoemission (PES) data for Bi_0.9_Sb_0.1_^6^. Here, the main bulk-like peak structure at 0.7 eV binding energy is resolved within LDA + DMFT.

To elucidate the crucial role played by local quantum correlations on electric transport in this *A*7 element, we show in the main panel of Fig. 3 the *T* dependence of dc resistivity computed using the orbital resolved, LDA + DMFT spectral functions, 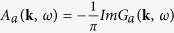. Within the Kubo formalism^52^, the dc-conductivity can be expressed as 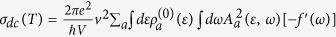, where 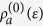 is the LDA DOS of the three (*x*, *y*, *z*) 6*p*-bands, 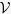 is the unit cell volume, and *f*(*ω*) is the Fermi function. As in ref. 53 the approximation made here is to ignore the **k**-dependence of electron’s velocity, *v*_*k*,*a*_. In this situation, following Saso *et al.*^54^,^55^, we approximate *v*_*k*,*a*_ by a single average carrier velocity (*v*) for all orbitals. In fact, Saso *et al.* and Baldassare *et al.*^56^ have shown that this assumption works well for numerical computations of *σ*_*ac*_(*ω*) for Kondo insulators (FeSi and YbB_12_) as well as for V_2_O_3_, supporting our approximation in *σ*_*dc*_(*T*) above. The observed features in resistivity *ρ*_*dc*_(*T*) ≡ 1/*σ*_*dc*_(*T*) originate from SO and Zeeman-field induced spectral changes: Showing how this provides a compelling description of extant experimental data is our focus here.

The dc resistivity in Fig. 3 shows metallic behaviour consistent with reconstructed spectral functions. Interestingly, for *V* = 1.05 eV we obtain a quasilinear *T*-dependence above 30 K which is close to that observed in bulk crystals^6^. Deviations between theory and experiment are observed below 30 K: Our result shows tendency towards saturation down to 9 K and a substantial drop to smaller resistivity values at low-*T*. Interestingly, this drop in resistivity at low-*T* is enhanced with increasing the SO interaction *V*, see bottom-right panel of Fig. 3. This electrical transport behaviour is in good semiquantitative agreement with resistivity data of bismuth single crystals^18^, lines^17^ and thin films^57^, all showing similar trend as found for 1.15 eV ≤ *V* ≤ 1.25 eV. In our theory this robust phenomenon is the fingerprint of SO-induced local quantum fluctuations^44^ in dc transport of elemental *p*-band semimetals like bismuth and graphite^29^. Taken together our theory-experiment comparison in Fig. 2 and the good semiquantitative agreement with resistivity data^6^ for *U* = 10 eV supports our proposal of non negligible electron-electron interactions in bismuth. In Fig. 3 we present additional evidence supporting this statement: We make a direct comparison between the LDA + DMFT spectra and PES data of Bi_0.9_Sb_0.1_ 6, showing good semiquantitative accord with experiment. Interestingly, the LDA + DMFT spectra resolves the main peak structure of bulk states^6^ at 0.7 eV binding energy in spite of the fact that the Bi_0.9_Sb_0.1_ system might exhibit some sort of lattice disorder effects induced by antimony. While PES experiment resolves a low-energy bump which was interpreted as gapless surface states coexisting with an insulating bulk^6^, the total LDA + DMFT spectral function shows a low-energy peak structure at −73 meV, in accordance with angle-resolved PES (ARPES) data reported in ref. 58 where it is found at 80 meV binding energy. Thus, our results demonstrate that low- and intermediate-energy features are well accounted for by bulk electronic structure. However, as seen in Fig. 2 the *p*_*z*_ DOS unveil a quasiparticle peak structure near *E*_*F*_, which is consistent with observation of out-of-plane surface states in (AR)PES^6^,^58^. Future experimental verification of *p*_*z*_ orbital symmetry in surface-like states would place our theory in a solid ground.

**Figure 4:**
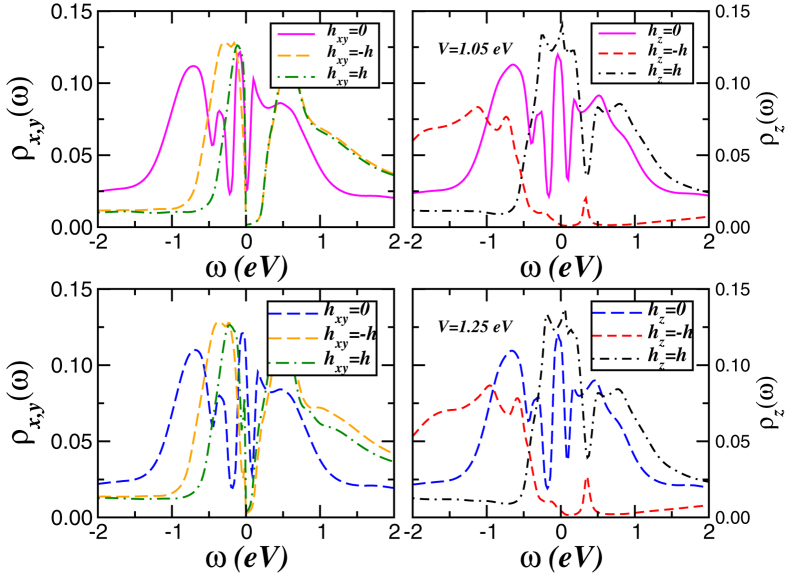
Zeeman field effects. Orbital-resolved LDA + DMFT DOS for magnetic fields applied parallel (*h*_*x*,*y*_) and perpendicular (*h*_*z*_) to the hexagonal plane. Top (bottom) panels show results for *V* = 1.05 eV (*V* = 1.25 V). Notice the field-induced Dirac valley^12^ in the basal *x*, *y*-plane DOS and the spin-selective insulating-metallic states in the out-of-plane (*p*_*z*_) orbital. The interplay between various competing effects in bismuth is visible.

We now turn to the interplay between local transverse-field fluctuations induced by SO coupling and intra-orbital Zeeman level splittings upon application of an external magnetic field in bismuth. To proceed, we consider the orbital-dependent on-site energy term, 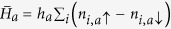 in our MO Hamiltonian, 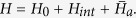 Here, 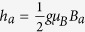, *μ*_*B*_ is the Bohr magneton, *g* is the Landé factor, and *B*_*a*_ is the external (Zeeman) magnetic field applied along the *a* direction^59^. Noteworthy, in our self-consistent treatment we vary the (trial) magnetic field *h*_*a*_, keeping the total band filling (*n*_*t*_ = 3) fixed to simulate the electronic changes upon Zeeman field splitting. As seen in Fig. 4, the variation in *h*_*a*_ drives appreciable SWT, producing drastic orbital-selective renormalisations of the one-particle spectral functions: The out-of-plane *p*_*z*_ band is most severely affected. As expected, *h*_*a*_ shifts the relative positions of the spin polarised bands at which the magnetism changes. The resulting magnetisation can be directly calculated from *m*_*a*_ = 〈*n*_*a*↑_〉 − 〈*n*_*a*↓_〉, with the particle’s number 〈*n*_*aσ*_〉 being computed using orbital and spin resolved spectral functions. According to our results a trial field applied along the hexagonal plane, i.e *h*_*x*,*y*_ = *h* (and *V* = 1.05 eV, 1.25 eV) gives negligibly small magnetisation values, *m*_*x*,*y*_ ≈ 0.01. This is an indication of reduced magnetic moments in the frustrated hexagonal plane. Interestingly, in this regime the in-plane Zeeman field drives the system towards to a Kondo insulating state with emergence of a narrow semiconducting band gap in the spin resolved spectral functions. Our results are thus consistent with a Dirac valley scenario in bulk bismuth^12^,^60^, which intriguingly implies a field-induced orbital reconstruction of the SO Kondo state into a Dirac liquid regime^61^. Moreover, the fact that the band gap is smaller for *V* = 1.25 eV compared to *V* = 1.05 eV can be regarded as the manifestation of SO induced weak antilocalisation effects^62^ in this and related (topological) quantum systems. Somewhat surprisingly are the Zeeman field effects on the *p*_*z*_ spectral functions. Here, we obtain *m*_*z*_ = 0.14 which arises from the formation of local moments in the majority (spin-↑) channel. As seen in the right panels of Fig. 4, bismuth undergoes a phase transition with an unusual two-fluid electron channel. While the majority *p*_*z*_-band is pushed towards to a Mott insulating state with appearance of a lower Hubbard band (local moment formation), the minority (↓) channel undergoes to a Kondo-like metallic state characterised by a broad quasiparticle peak at *E*_*F*_. This unexpected field-induced phase transition is apparently consistent with recent DMFT study^59^, showing coexisting metallic and insulating states in the spin resolved spectral functions of a spin-asymmetric Hubbard model. As a major result, we thus predict that destroying the Kondo metal state (Fig. 4 right panels) by a moderate number of impurities, vacancies or lattices defects (all inducing one-electron band narrowing) will reveal large MR behaviour as in experiment. We emphasise that resistivity measurements in strained bismuth are an alternative test to our proposal. This would reduce the quasi-coherent Kondo scale of the low-energy peak in the spin-selective state in Fig. 4, promoting large resistivity responses currently unreachable. More detailed theoretical and experimental work to establish or refute our prediction are thus called for.

## Discussion

**Figure 5:**
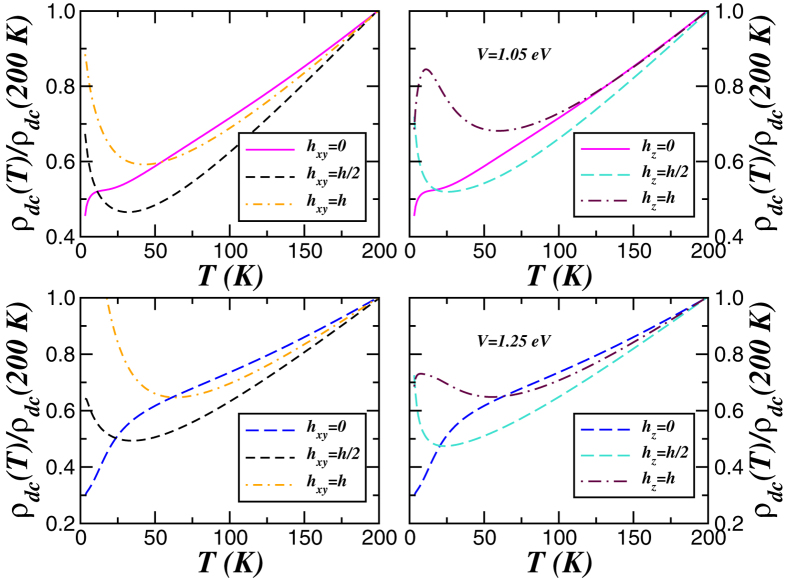
Magnetoresistance. Temperature dependence of dc resistivity of bismuth for Zeeman fields applied parallel (*h*_*x*,*y*_) and perpendicular (*h*_*z*_) to the basal, hexagonal plane. Side panels show results for two values of local spin-orbit interaction, *V* = 1.05 (top) and *V* = 1.25 (bottom). Noteworthy, our results are in good semiquantitative agreement with magnetoresistance data reported experimentally^11^,^12^,^17^,^18^.

An understanding of the correlated nature of *p*-band semimetals is mandatory for designing novel MR materials. An affirmative theoretical answer to the MR effect in elemental bismuth is shown in Fig. 5. In this figure we display the dc resistivity [*ρ*_*dc*_(*T*)] computed using LDA + DMFT spectral functions within the Kubo formalism^52^. In our Green’s-function formalism the observed features in the directional-selective^12^ resistivity responses originate from the spectral changes with Zeeman field, *h*_*a*_: Showing how this provides a compelling explanation for the MR effect for in-plane (*h*_*x*,*y*_) and out-of-plane (*h*_*z*_) magnetic fields is our focus here. Various interesting features immediately stand out in Fig. 5. First, as *T* → 0 the resistivity curve for a trial field 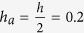 eV/*μ*_*B*_^59^ shows semiconducting-like *T*-dependence, in accordance with the insulating behaviour at small fields seen in experiment^18^. Our choice is also consistent with initial magnetic field values used to produce differences between spin flavours within DMFT in systems with narrower bandwith^63^. Second, with increasing *h*_*a*_ the dc resistivity in Fig. 5 shows orbital-selectivity manifesting itself as directional selectivity. The left panels of Fig. 5 display our results for field applied parallel to the hexagonal (basal) plane of bulk bismuth. As seen, below 50 K *ρ*_*dc*_(*T*) increases as *T* → 0 at finite in-plane field, *h*_*x*,*y*_, which is consistent with a semiconducting like behaviour due to field-induced Dirac valley spectrum^12^. Further interesting features emerge for fields applied perpendicular to the basal plane where we find low-*T* metallicity accompanying an insulator-to-metal crossover. The interplay between local spin fluctuations^44^ and magnetic field-induced band splitting thus promotes a smooth crossover to low-energy quasi-coherence by internally generating a metallic channel that locally switches on the recoil process in the minority-spin channel: If this had not occurred, one could have a multi-orbital Kondo insulator^27^,^28^ down to lowest *T*. This is our mechanism for the experimentally observed reduction of *ρ*_*dc*_(*T*) at low *T* in bismuth, where a crossover from a metal to an insulating-like behaviour above *T* = 10 K is obtained, in good semiquantitative agreement with extant data^11^,^17^. It is interesting that a LDA + DMFT calculation is able to access the emerging MR anisotropy at different magnetic fields^12^ in this *p*-band semimetal.

## Conclusions

In conclusion, using LDA + DMFT for a multi-band model, we resolve the nature of the metallic regime in bismuth. Good semiquantitative accord with key spectral^6^,^30^ and transport data^6^ in a quantum correlated picture confirms that charge carriers in elemental Bi interact via Coulomb repulsion^25^. The Zeeman coupling induces two distinct electronic transitions characterised by selective Kondo-Mott physics. Although the bare applied field is globally uniform, the effective field experienced by the *p* electrons differs because of various competing local quantum fluctuations. For fields applied parallel to the hexagonal plane the emergent Dirac valley reflects the competition between Zeeman field splitting and screening of local moments. On the other hand, for fields perpendicular to the basal plane spin-selective metallic and insulating states similar to that of correlated half-metals^59^ are effectively promoted. In this unconventional regime with coexisting Kondo-Mott low-energy physics, the resistivity profiles found here are in good semiquantitative agreement with magnetoresistance responses in the experimentally important case in which the orientation of the in-plane magnetic field^12^ does not break the intrinsic orbital degeneracy of bulk bismuth. Our microscopic description of competing multi-orbital interactions is expected to be generally applicable to partially filled *p*-band semimetals^18^,^64^,^65^ of pivotal importance.

## Methods

The local-density-approximation plus dynamical-mean-field-theory (LDA + DMFT) by construction takes into consideration the most relevant multi-orbital correlation effects and all-electron degrees of freedom. It is an ideal starting point towards the description of Coulomb-driven metal-to-insulator transitions, Fermi and non-Fermi liquid metallic states and in general grounds the role played by dynamical correlations in idealised many-particle models as well as in real multi-orbital systems^26^. The LDA + DMFT is a theoretical tool which provides realistic answers to interesting questions like why spin, orbital and magnetic orders in strongly correlated electron systems set in at low temperatures and how they might change upon application of external perturbations like pressure, chemical doping, magnetic and electric fields, etc. Noteworthy, the many-particle DMFT scheme^66^, without realistic band structure inputs, is an exact theory when approaching the limit of high lattice dimensions^67^. It is designed to describe the time evolution (i.e., dynamical effects) of local spin and charge fluctuations^68^, intrinsic to correlated electron systems.

In order to prove the orbital-selective nature of the *p*-band electronic states in elemental bismuth, we employ our state-of-the-art implementation of LDA + DMFT^46^, which correctly takes disorder, temperature and pressure effects into account, in multi-band systems. The one-particle, LDA density-of-states are computed using the non-fully relativistic version of the PY-LMTO code^42^. Self-consistency is reached by performing calculations with 417 irreducible **k**-points. The radii of the atomic spheres were chosen as *r* = 3.35 a.u. in order to minimise their overlap. To incorporate the effects of electronic correlations in this *p*-band semimetal, we use the multi-orbital iterated-perturbation-theory (MO-IPT) as an impurity solver of the many-particle problem in DMFT, as described in detail in refs. 45,46. Finally, we carried out the computation of electrical transport within the Kubo formalism^52^.

A Hubbard *U* = 10 eV was necessary for a good semi-quantitative agreement with experiments within LDA + DMFT, see Fig. 2 (right-lower panel) and Fig. 3. For main group elements, values of *U* larger than typical values for transition metals are the consequence of orbital hybridisation and covalent bonds, which localise electrons between atoms^35^,^69^. To confirm the role of electronic correlation in elemental bismuth, we have also performed LDA + U calculations based on the PY-LMTO code^42^. Unlike standard DFT calculations, finding the ground state in LDA + U may depend on initial orbital occupancy. Performing unbiased LDA + U (*U* ≤ 12), orbital occupancy yields *n*_*x*,*y*_ = *n*_*z*_ = 1.0. Biasing *n*_*x*,*y*_, LDA + U calculations (*U* ≈ 10 eV) converge towards the ground state with *n*_*x*,*y*_ = 1.05, *n*_*z*_ = 0.9 in good agreement with LDA + DMFT.

## Additional Information

**How to cite this article**: Craco, L. and Leoni, S. Magnetoresistance in the Spin-Orbit Kondo State of Elemental Bismuth. *Sci. Rep.* **5**, 13772; doi: 10.1038/srep13772 (2015).
